# Supplementary material for: MEIS2 Is an Adrenergic Core Regulatory Transcription Factor Involved in Early Initiation of TH-MYCN-Driven Neuroblastoma Formation
Source: Cancers (Basel). 2021 Sep 24;13(19):4783. doi: 10.3390/cancers13194783 (PMC8508013; doi:10.3390/cancers13194783)
Supplement: Supplementary file 1 [file cancers-13-04783-s001.zip › cancers-1386380-supplementary.pdf]

Supplementary Materials: MEIS2 Is an Adrenergic Core Regulatory Transcription Factor Involved in Early Initiation of TH-MYCN-Driven Neuroblastoma Formation

Jolien De Wyn, Mark M. Zimmerman, Nina Weichert-Leahey, Carolina Nunes, Belamy B. Cheung, Brian J. Abraham, Anneleen Beckers, Pieter-Jan Volders, Bieke Decaesteker, Daniel R. Carter, Alfred Thomas Look, Katleen De Preter, Wouter Van Loocke, Glenn M. Marshall, Adam D. Durbin, Frank Speleman and Kaat Durinck

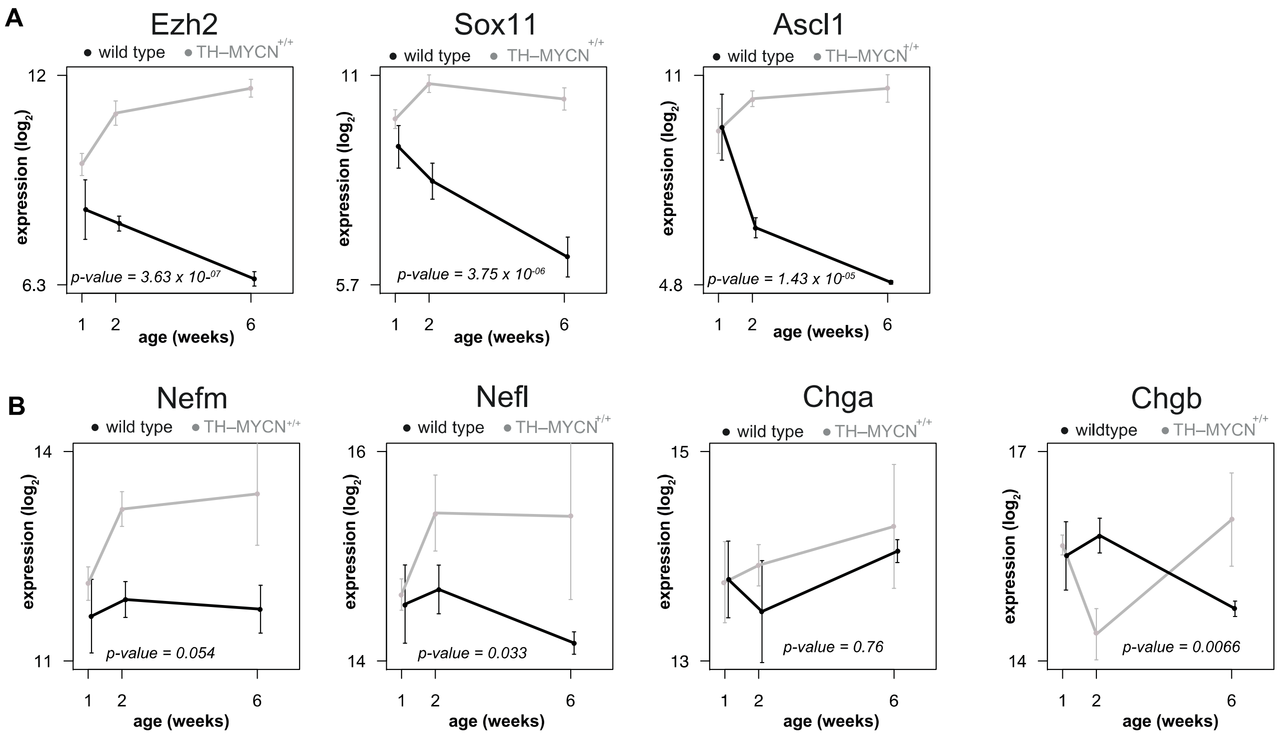

Figure S1. Time-resolved expression patterns of immature and mature adrenergic makers during murine TH-MYCN driven neuroblastoma tumor development. (**A**) Expression of *Ezh2*, *Sox11* and *Ascl1* as part of the immature adrenergic geneset in the TH-MYCN dataset; (**B**) Expression of *Nefm*, *Nefl*, *Chga* and *Chgb* as part of the mature adrenergic geneset in the TH-MYCN dataset. The data shown present mean gene expression ± standard deviation of 4 samples. *grey*: TH-MYCN$^{+/+}$ samples; *black*: wild type samples. *p*-value corresponds to the interaction term of a two-way ANOVA analysis.

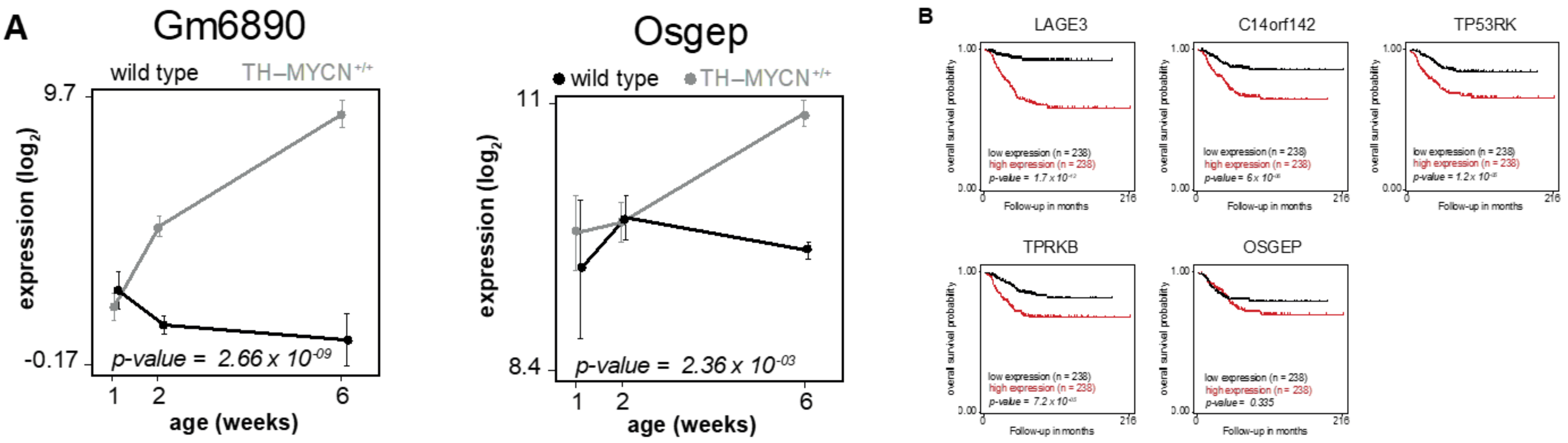

Figure S2. Time-resolved expression patterns of immature and mature adrenergic makers during murine TH-MYCN driven neuroblastoma tumor development. (**A**) Expression of *Gm6990* and *Osgep*, both part of the KEOPS complex, in the dataset. The data shown present mean gene expression ± standard deviation of 4 samples. *grey*: TH-MYCN$^{+/+}$ samples; *black*: wild type samples. *p*-value corresponds to the interaction term of a two-way ANOVA analysis; (**B**) Kaplan-Meier analysis of overall patient survival based on gene expression levels of *LAGE3, C14orf142, TP53RK, TPRKB* and *OSGEP*, components of the KEOPS complex, in a large cohort of 649 primary neuroblastoma patients (GSE45547, *n* = 649) with high or low expression using median as a cut-off. *p*-values are a result of a log-rank test.
